# Prevalence and risk factors associated with self-reported psychological distress among college students during the omicron outbreak in Shanghai

**DOI:** 10.3389/fpubh.2022.936988

**Published:** 2022-07-22

**Authors:** Wei Li

**Affiliations:** ^1^Department of Geriatric Psychiatry, Shanghai Mental Health Center, Shanghai Jiao Tong University School of Medicine, Shanghai, China; ^2^Alzheimer's Disease and Related Disorders Center, Shanghai Jiao Tong University, Shanghai, China

**Keywords:** Omicron, anxiety, depression, prevalence, college students

## Abstract

**Background:**

More and more attention has been paid to the mental health of students in higher education. The Omicron outbreak has brought renewed attention to this vulnerable group.

**Objective:**

To understand the prevalence and influencing factors of anxiety symptoms and depression symptoms of college students in a closed state.

**Methods:**

This large cross-sectional study using data from a survey on the mental health of college students in Shanghai (China), conducted by using a stratified cluster random sampling method between March 15th and April 15th, 2022. To estimate results related to regional location, only data from students with Internet protocol addresses and current addresses in Shanghai were included. The main outcome was self-reported psychological distress (including depressive symptoms, anxiety symptoms, and self-assessment of health), measured using the epidemiologic studies depression scale (CES-D), the Spielberger state-trait anxiety inventory (STAI) and self-rated mental health (SRMH), respectively. Moreover, the Simplified Coping Style Scale (SCSS) was also used to assess how participants coped with negative emotions.

**Results:**

Among 13,000 college students who completed the survey, 12,124 students were included in the final analysis, and the total effective rate was 93.3%. The prevalence of depressive symptoms and anxiety symptoms were 14.1 and 9.8%, respectively. By using Multivariate logistics regression analysis, we found that being male and negative coping were risk factors for depressive symptoms and anxiety symptoms, while positive coping, such as study or learning, were protective factors. Moreover, linear regression analysis showed that learning or study improved the overall mental health index by improving anxiety or depressive symptoms, and played a partial mediating role.

**Conclusions:**

These findings suggest that a significant number of college students, especially boys, will experience emotional problems during the course of closed schools. Therefore, we need to give them proper attention and advise them to adopt positive coping strategies, such as learning or study, to resist bad emotions.

## Introduction

COVID-19 is a novel acute respiratory infectious disease caused by the infection of novel Coronavirus (2019-COV-2). On 26 November 2021, the World Health Organization (WHO) announced a new 2019-COV-2 variant Omicron (B.1.1.529) (1). Despite its apparent decline in virulence, Omicron is becoming more infectious, thus posing new challenges to epidemic control (2). Prevention and control of COVID-19 remains a public health priority worldwide, and China has adjusted a number of policies to contain the spread of Omicron, such as city closures and travel restrictions (3). Although the above measures are effective, the ongoing epidemic and burdensome measures such as lockdown and stay-at-home orders can also cause certain psychological problems. For example, Choi et al. found that during the omicron epidemic in Hong Kong, the prevalence of anxiety and depression among the general population was 19 and 14% (4), respectively.

Since college students live in groups, there is no doubt that they are a vulnerable group in COVID-19. The inconvenience of life, inability to complete their studies caused by school closures as well as the worry and fear of COVID-19 may also have a certain impact on their psychological and mental state. For example, Chang et al., found that the overall incidence of anxiety and depressive emotions among college students during the COVID-19 outbreak were 26.60 and 21.60%, respectively (5). Fu et al. found that about 41.1% of Chinese college students experienced anxiety symptoms during the COVID-19 epidemic (6). While in Ma et al.' study, they found that the prevalence rates of probable acute stress, depressive and anxiety symptoms among Chinese college students during the COVID-19 outbreak were 34.9, 21.1, and 11.0%, respectively (7).

Since isolation at school is more inconvenient, more susceptible to infection and more vulnerable to adverse external information than isolation at home, our hypothesis is that school isolation may have a more serious psychological impact on college students. Therefore, we carried out this large-scale survey to focus on the school isolation effects on college students' mental state.

## Materials and methods

### Participants

**Figure 1:**
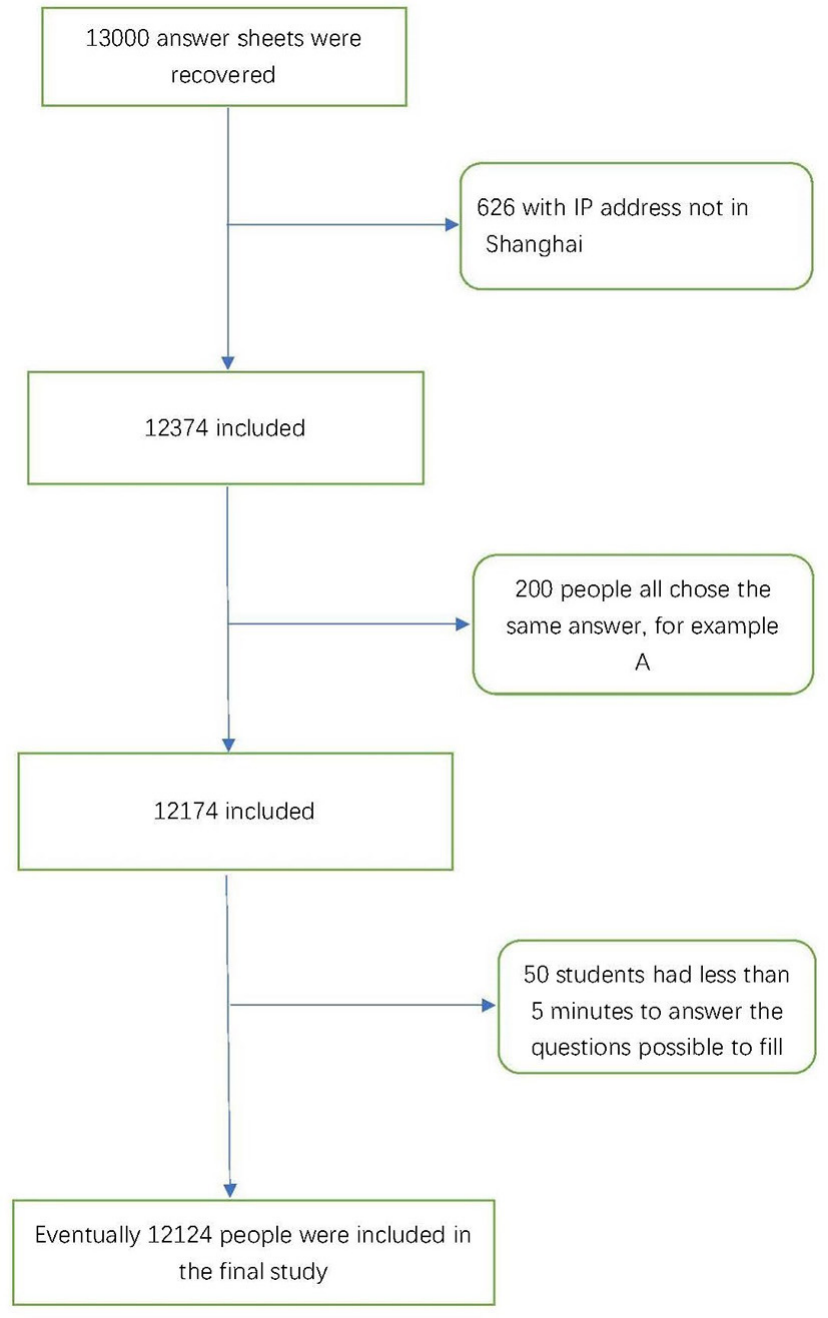
A flow chart of the entire study.

From March 15th, 2022, to April 15th, 2022, the cluster sampling was used to survey college students (including undergraduate, graduate and doctoral students) over the age of 18 in Shanghai. Structured questionnaires (online) were used to assess the mental health of these students during the Omicron outbreak. The questionnaires were anonymous to ensure the confidentiality and reliability of the data. Inclusion criteria included: (1) age 18 and above; (2) have a smartphone; (3) university education in the Shanghai area; (4) under quarantine at school. Exclusion criteria were as follows: (1) not in Shanghai; (2) non-enrolled students; (3) pre-existing anxiety or depression symptoms; (4) under quarantine at home or elsewhere. Finally, A total of 13,000 college students were given questionnaires (including Shanghai Jiao Tong University, Fudan University, East China University of Science and Technology and other universities, the main way of diffusion was wechat moments forwarding), and 12,124 valid questionnaires were returned, with a total effective rate of 93.3%. Figure 1 lists the research process of the whole study.

Ethical approval was issued by the Ethics Committee of Shanghai Jiao Tong University, and all the participants had given informed consent before the study was initiated.

### Assessment instruments

The study instrument comprised a structured questionnaire that included demographic information (gender, grade, age, special field of study, marital status, only child, and family monthly income) and information about their cognition and preventive behaviors regarding Omicron. Moreover, the participants responded to the Center for Epidemiologic Studies Depression Scale (CES-D), the Spielberger State-Trait Anxiety Inventory (STAI), the Simplified Coping Style Scale (SCSS) and self-rated mental health (SRMH).

#### The center for epidemiologic studies depression scale

The Center for Epidemiologic Studies Depression Scale (CES-D) was used to assess depression symptoms (8). It is a self-assessment scale of 20-item with validity of 0.9, reliability of 0.67, each item is rated using a 4-point Likert scale to represent how frequently the symptom occurred in the past week (0 = rarely/ <1 day, 1 = sometimes/1–2 days, 2 = often/3–4 days, 3 = most of the time/5–7 days). The score range of CES-D is 0–60, with higher scores indicating a greater degree of depression symptoms. Generally, a cut-off score of ≥16 is considered to have clinically meaningful depressive symptoms (9).

#### The spielberger state-trait anxiety inventory

The Spielberger State-Trait Anxiety Inventory (STAI) is a 40-item self-report measure of anxiety using a 4-point Likert-type scale (from 0 to 3 points) for each item. It has two scales: State anxiety and Trait anxiety. Both scales consist of 20 items. The study chooses the State Anxiety Scale (S-Anxiety) evaluates immediate or recent experiences or feelings of fear, tension, anxiety, and neuroticism at a particular time or situation and can be used to evaluate state anxiety in stressful situations (10). Responses to the S-Anxiety scale measure the frequency of feelings “in general”: (1) almost always, (2) often, (3) sometimes, and (4) almost never. The range of scores for S-Anxiety is 20–80, and the higher score indicates greater anxiety symptoms (11). So we used 45.13 as the cut-off value to determine whether the subjects had anxiety (12).

#### The simplified coping style questionnaire

In 1998, Hu et al. revised the Simplified Coping Style Questionnaire (SCSQ) on the basis of the Ways of Coping Questionnaire (Folkman) (13). SCSQ is a 20-item self-reported questionnaire that includes two dimensions: active coping (12 items) and passive coping (8 items). Responses are asked to provide on a 4-point scale according to how frequently respondents adopt each item, from 1 “never” to 4 “very often”. The higher scores represent the greater positive and negative coping styles (14). Previous studies have shown that the Cronbach's α of the SCSS scale is 0.90, which suggests its high reliability and validity (15).

#### The self-rated mental health

Self-Rated Mental Health (SRMH) can be used to measure symptoms associated with psychiatric disorders and psychological distress. Responses are asked “How will you rate your overall mental health?” Responses will include five categories: poor, fair, good, very good and excellent. In a short time interval, the test retest reliability range is 0.7–0.8, indicating that the single-question retest reliability is high (16). The higher the SRMH score, the better the mental state of the subject. Moreover, previous studies have also confirmed that SRMH is equally effective across ethnic groups (17).

### Demographic characteristics

Age was defined as a categorical variable with three groups: <18, 18–29, or 30 years or older (their average age was 19.35, 95% confidence interval: 18.71–24.37). Gender was defined as a binary variable for male or female. An only child was defined as having only one child in a family. Learning professional was defined as a categorical variable with 5 groups: humanities and social science, institute of technology, art or sports, medical or others. Grade was defined as a categorical variable with five groups: grade 1, grade 2, grade 3, grade 4, and grade 5. Study was defined as spending most of the day reading or reviewing professional knowledge, while Play gaming was defined as spending most of the day playing mobile phone or computer games.

## Statistical analysis

Descriptive statistics were used to illustrate the demographic and other selected characteristics of the college students. Continuous variables were presented as the mean (SD), while categorical variables were presented as the frequency (%). The one-sample Kolmogorov-Smirnov test was used to test whether the data conformed to normal distribution. The Mann-Whitney *U*-test was used to compare the continuous variables without normal distribution, and the Chi-square test was used to compare categorical variables. Statistically significant variables were screened and included in multivariate logistic regression analyzes (treating the presence of depressive symptoms or anxiety symptoms as a dependent variable, for depressive symptoms, the independent variables included age, male, grade, SRMH, positive coping, negative coping, study and play games; for anxiety symptoms, the independent variables included male, grade, SRMH, positive coping, negative coping, study and play games). Correlation analysis was used to explore the relationship between neuropsychological tests. Moreover, Linear regression model was used to investigate the relationship between SRMH total score, study, CES-D and STAI total score [The regression equation was: (1) Y = CX + e1, (2) M = AX + e2, (3) Y = c'X + bM + e3; in model 1, the coefficient C was the total effect of X on Y; in model 2, the coefficient A was the direct effect of X on M; in model 3, The coefficient B was the direct effect of M on Y after controlling the influence of X; The coefficient C 'was the direct effect of X on Y after controlling the influence of M; The coefficient a^*^b was the mediating effect produced by the mediating variable M, and there was a relationship between a^*^b = C–C ']. All the statistical analysis was performed using SPSS version 22.0 and a *p*-value <0.05 was considered as significant.

## Results

### Levels of depressive symptoms and anxiety symptoms among college students during the epidemic

Of the 12,124 college students, 1,705 (14.1%) had significant depressive mood, while 1,191 (9.8%) showed significant anxiety symptoms.

### Factors influencing college students' depressive symptoms and anxiety symptoms during the epidemic (univariate analysis)

Using non-parametric or Chi-square tests, we found that age, gender, grade level, SRMH, positive coping, negative coping, study and play games had significant effects (*p* < 0.05) on depression, while being an only child, and learning professional were not associated with depression symptoms (*p* > 0.05). Similarly, gender, grade level, SRMH, positive coping, negative coping, study and play games had significant effects (*p* < 0.05) on anxiety, while age, being an only child and learning professional were not associated with anxiety symptoms (*p* > 0.05). Table 1 presents the results.

**Table 1:** Univariate and multivariate analysis of participants with and without depression or anxiety symptom.

**Variables**	**CES-D**					**STAI**				
	**Univariate analysis**			**Multivariate analysis**		**Univariate analysis**			**Multivariate analysis**	
	**Depression (*****n*** = **1,705)**	**Non-depression (*****n*** = **10,419)**	***p***	**Adjusted OR (95% CI)**	***P***	**Anxiety (*****n*** = **1,191)**	**Non-anxiety (*****n*** = **10,933)**	***p***	**Adjusted OR (95% CI)**	***p***
Age										
<18 years old, *n* (%)	23 (1.3)	121 (1.2)	0.026*	0.090 (0.006–1,258)	0.074	14 (1.2)	130 (1.2)	0.392	–	–
18–29 years old, *n* (%)	1,680 (98.5)	10,297 (98.8)		0.079 (0.006–1.048)	0.054	1,176 (98.7)	10,801 (98.8)			
>30 years old, *n* (%)	2 (0.1)	1 (0)		–	–	1 (0.1)	2 (0)			
Male, *n* (%)	955 (56.0)	4,576 (43.9)	<0.001*	1.503 (1.329–1.700)	<0.001*	620 (52.1)	4,911 (44.9)	<0.001*	1.239 (1.080–1.420)	0.002*
An only child, *n* (%)	534 (31.3)	3,025 (29.0)	0.058	–	–	342 (28.7)	3,217 (29.4)	0.638	–	–
Learning professional										
Humanities and social science	109 (6.4)	723 (6.9)	0.064	–	–	61 (5.1)	771 (7.1)	0.131	–	–
Institute of technology	957 (56.1)	5,709 (54.8)				669 (56.2)	5,997 (54.9)			
Art or sports	66 (3.9)	546 (5.2)				61 (5.1)	551 (5.0)			
Medical	424 (24.9)	2,443 (23.4)				278 (23.3)	2,589 (23.7)			
Others	149 (8.7)	998 (9.6)				122 (10.2)	1,025 (9.4)			
Grade										
Grade one	440 (25.8)	3,200 (30.7)	<0.001*	0.946 (0.635–1.411)	0.787	276 (23.2)	3,364 (30.8)	<0.001*	0..881 (0.565–1.375)	0.577
Grade two	417 (24.5)	2,711 (26.0)		0.982 (0.659–1.465)	0.930	328 (27.5)	2,800 (25.6)		1.154 (0.741–1,795)	0.526
Grade three	415 (24.3)	2,367 (22.7)		1.022 (0.685–1.525)	0.915	296 (24.9)	2,486 (22.7)		1.147 (0.736–1,789)	0.544
Grade four	391 (22.9)	1,922 (18.4)		1.131 (0.757–1.690)	0.547	262 (22.0)	2,051 (18.8)		1.139 (0.729–1,779)	0.569
Grade five	42 (2.5)	219 (2.1)		–	–	29 (2.4)	232 (2.1)		–	–
SRMH	87.45 (13.12)	107.28 (23.18)	<0.001*	0.948 (0.944–0.952)	<0.001*	90.53 (15.69)	106.01 (23.26)	<0.001*	0.966 (0.962–0.970)	<0.001*
Positive coping	16.51 (5.61)	19.58 (6.97)	<0.001*	0.891 (0.880–0.901)	<0.001*	19.45 (5.63)	19.11 (7.00)	0.001*	0.979 (0.967–0.990)	<0.001*
Negative coping	10.34 (3.52)	7.12 (4.02)	<0.001*	1.296 (1.273–1.319)	<0.001*	11.51 (4.05)	7.14 (3.88)	<0.001*	1.299 (1.275–1.323)	<0.001*
How to arrange the time										
Study, *n* (%)	1,227 (72.0)	8,706 (83.6)	<0.001*	0.862 (0.751–0.990)	0.035*	886 (74.4)	9,047 (82.7)	<0.001*	0.796 (0.680–0.931)	0.004*
Play games, *n* (%)	1,021 (59.9)	5,431 (52.1)	<0.001*	1.114 (0.984–1.261)	0.089	680 (57.1)	5,772 (52.8)	0.005*	1.015 (0.885–1.166)	0.828

In the regression model of depressive symptoms, the existence of depressive symptoms is taken as a dependent variable, and the included independent variables include age, male, grade, going out to reduce, wearing a mask, wash hands more often, Frequent disinfection, Adjust travel time, study, play phone, and play games; In the regression model of anxiety symptoms, the existence of anxiety symptoms is taken as a dependent variable, and the included independent variables include male, grade, going out to reduce, wearing a mask, wash hands more often, Adjust travel time, study, play phone, and play games; CES-D means the Center for Epidemiologic Studies Depression Scale; STAI means the Spielberger State-Trait Anxiety Inventory; SRMH means the Self-Rated Mental Health; *means p <0.05.

### Factors influencing college students' depressive symptoms and anxiety symptoms during the epidemic (multivariate analysis)

**Figure 2:**
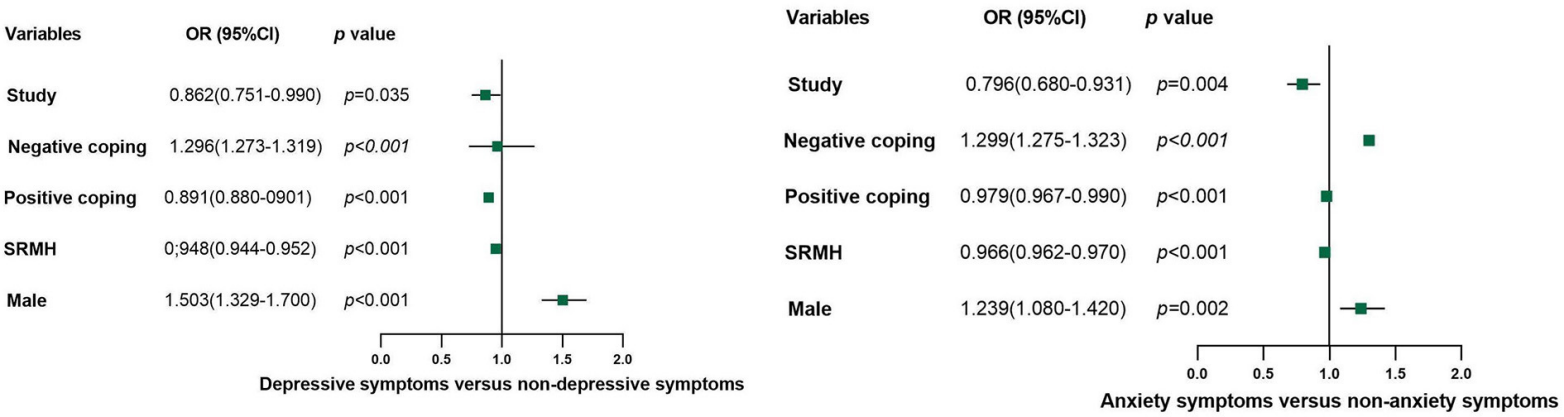
Influencing factors that may be associated with depressive symptoms and anxiety symptoms.

By using multiple logistics regression analysis and taking the presence of depressive symptoms or anxiety symptoms as the dependent variable, we found that SRMH (*p* < 0.001, OR = 0.948, 95%confidence interval: 0.944–0.952), positive coping (*p* < 0.001, OR = 0.891, 95%confidence interval: 0.880–0901), study (*p* = 0.035, OR = 0.862, 95%confidence interval: 0.751–0.990) were protective factors for depressive symptoms, while male (*p* < 0.001, OR = 1.503, 95%confidence interval: 1.329–1.700) and negative coping (*p* < 0.001, OR = 1.296, 95%confidence interval: 1.273–1.319) were risk factors. Similarly, we also found that SRMH (*p* < 0.001, OR = 0.966, 95%confidence interval: 0.962–0.970), positive coping (*p* < 0.001, OR = 0.979, 95%confidence interval: 0.967–0.990), study (*p* = 0.004, OR = 0.796, 95%confidence interval: 0.680–0.931) were protective factors for anxiety symptoms, while male (*p* = 0.002, OR = 1.239, 95%confidence interval: 1.080–1.420) and negative coping (*p* < 0.001, OR = 1.229, 95%confidence interval: 1.275–1.323) were risk factors. Table 1 and Figure 2 present the results.

### Correlation analysis between neuropsychological tests

**Figure 3:**
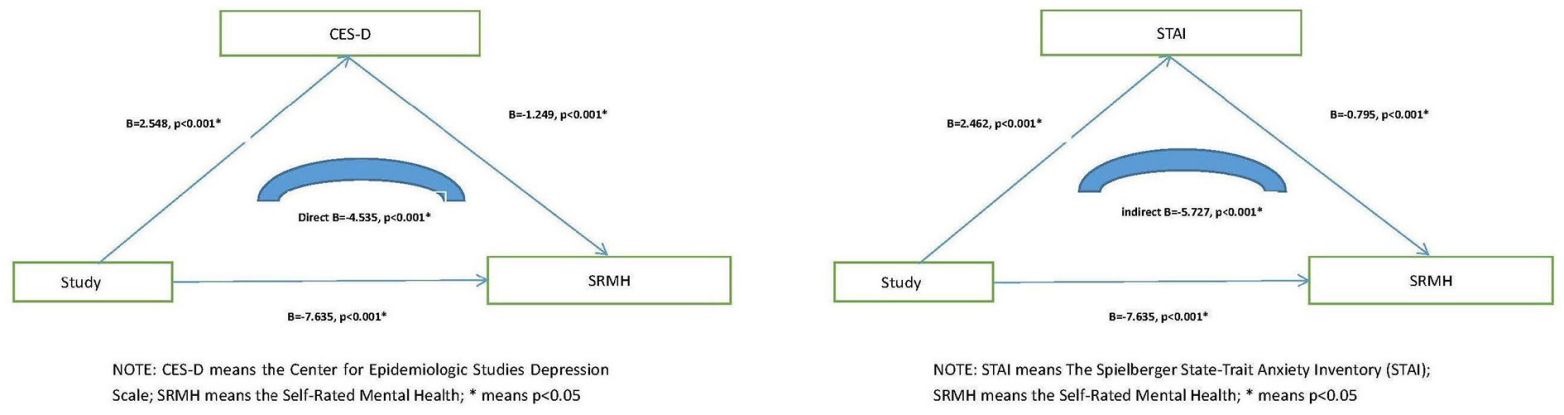
The relationship between SRMH total score, study, CES-D and STAI total score.

The results of correlation analysis showed that STAI was positively correlated with CES-D (*r* = 0.557, *p* < 0.001) and negative coping (*r* = 0.414, *p* < 0.001), but negatively correlated with positive coping (*r* = −0.073, *p* < 0.001) and SRMH (*r* = −0.352, *p* < 0.001). Moreover, we also found that CES-D was positively correlated with negative coping (*r* = 0.350, *p* < 0.001), but negatively correlated with positive coping (*r* = −0.191, *p* < 0.001) and SRMH (*r* = −0.395, *p* < 0.001). These results suggested that anxiety and depression symptoms were negatively correlated with the health self-rating scale, and a positive coping style would help to prevent anxiety and depression, while negative coping style might aggravate anxiety and depression. Table 2 presents the results. Moreover, linear regression model was used to investigate the relationship between SRMH total score, study, CES-D and STAI total score, and we found that study affected SRMH total score by influencing CES-D (B = −4.535, *p* < 0.001) and STAI total score (B = −5.727, *p* < 0.001), and played a partial mediation effect (the SRMH was taken as the dependent variable, study as the independent variable, the result of linear regression analysis showed that B = −7.635, *p* < 0.001; the CES-D was taken as the dependent variable, study as the independent variable, the result of linear regression analysis showed that B = −2.548, *p* < 0.001; then the SRMH was taken as the dependent variable, CES-D and study were treated as independent variables, respectively, the result of linear regression analysis showed that B = −4.535, *p* < 0.001; The relationship between SRMH and STAI and study was consistent with the previous analysis process). Figure 3 presents the results.

**Table 2:** Correlation between neuropsychological tests.

**Variables**	**Variables**	**STAI**	**CES-D**	**Positive coping**	**Negative coping**	**SRMH**
STAI	Pearson coefficient	1	0.557	−0.073	0.414	−0.352
	p		<0.001*	<0.001*	<0.001*	<0.001*
	N	12,124	12,124	12,124	12,124	12,124
CES-D	Pearson coefficient	0.557	1	−0.191	350	−0.395
	p	<0.001*		<0.001*	<0.001*	<0.001*
	N	12,124	12,124	12,124	12,124	12,124
Positive coping	Pearson coefficient	−0.073	−0.191	1	0.258	0.237
	p	<0.001*	<0.001*		<0.001*	<0.001*
	N	12,124	12,124	12,124	12,124	12,124
Negative coping	Pearson coefficient	0.414	0.350	258	1	−0.180
	p	<0.001*	<0.001*	<0.001*		<0.001*
	N	12,124	12,124	12,124	12,124	12,124
SRMH	Pearson coefficient	−0.352	−0.395	0.237	−0.187	1
	p	<0.001*	<0.001*	<0.001*	<0.001*	
	N	12,124	12,124	12,124	12,124	12,124

CES-D means the Center for Epidemiologic Studies Depression Scale; STAI means the Spielberger State-Trait Anxiety Inventory; SRMH means the Self-Rated Mental Health; *means p <0.05.

## Discussions

In this large, cross-sectional study, we explored the effects of the omicron outbreak on the psychological status of college students and drew several interesting conclusions: (1) the prevalence of depressive symptoms and anxiety symptoms among the Chinese college students was 14.1 and 9.8%, respectively; (2) being male is a risk factor for both depressive symptoms and anxiety symptoms; (3) depression and anxiety symptoms were negatively correlated with the health self-rating scale, and positive coping style (such as study) would help to prevent anxiety and depression, while negative coping style might aggravate anxiety and depression.

Previous studies have shown that isolation due to COVID-19 could take a toll on the psychological state of college students. For example, Yu et al., found that the prevalence of depressive symptoms was 15.8% (1,486/9,383) among Chinese college students (18). Ma et al. (7) found that the prevalence rates of depressive and anxiety symptoms among Chinese college students were 21.1 and 11.0%, respectively. In Guan et al.'s (19) study, they found that the overall prevalence of anxiety among Chinese college students was 7.3%, while in Fu et al.'s study, they found that 41.1% of Chinese college students experienced anxiety symptoms during the COVID-19 epidemic (6). By using CES-D and STAI, we found the prevalence of depressive symptoms and anxiety symptoms among Chinese college students was 14.1 and 9.8%, respectively. Therefore, the conclusions of different studies often vary greatly, which may be due to the use of different emotional symptom assessment scales with inconsistent sensitivity and specificity. In addition, the timing of the investigation might also affect the results, as those previous studies were carried out in the early stages of the Novel Coronavirus outbreak, when there was a great deal of fear and uncertainty about the disease. At the time of this study, many college students were already well aware of the disease, and the incidence of anxiety and depression may have decreased accordingly.

In our current study, we found that being male was a major risk factor for both anxiety symptoms and depressive symptoms, seemingly contrary to previous research. According to the world health organization (WHO), women are more likely to suffer from depression (5.1% compared to 3.6% worldwide) and anxiety (4.6% compared to 2.6% worldwide) than men (20). Previous studies have also shown that adolescent and young adult females are more prone to depression than males (21, 22). For example, Fawzy and Hamed (23) found that female sex was significantly associated with stress, depression and anxiety scores. And Qi et al. (24) found that the prevalence of psychotic depression (PD) in female patients (10.97%) was higher than that in male patients (7.99%). Therefore, our findings were contrary to those of others, and we hypothesized that male students were more active and socially inclined (in the face of bad emotions, male college students tend to face alone, or it was not easy to talk to friends like female college students), which might have a greater impact on their psychological state once they were confined to school. Moreover, compared with female college students, male college students were more likely to use negative and bad ways to deal with negative emotions (25). However, further exploration and verification were needed for the above research conclusions.

Moreover, we found that different ways of coping with emotions may have different outcomes. By using the simplified coping style questionnaire (SCSQ), we found that positive coping with emotional symptoms, such as learning or study, could effectively prevent anxiety and depression (learning or study could affect the overall mental health of individuals by improving anxiety or depression, and played a part of the mediating effect), while negative coping might increase the risk of anxiety or depressive symptoms. Zhang et al. found that positive coping was a protective factor for trauma-related distress in junior high school students, while negative coping was a risk factor (26). Si et al., found that passive coping strategies were positively correlated to Posttraumatic stress and depression, Anxiety and Stress Scale (DASS) scores (27). In Sun et al.'s study, they found that active guidance of psychological growth could promote physical and mental recovery in COVID-19 patients (28). In Zhao et al.'s (29) study, they found that aerobic exercise, resistance exercise, and mind-body exercise could improve depressive symptoms and levels. Moreover, Xiong et al. also found that higher negative coping style scores would increase the prevalence of anxiety symptoms (30). Therefore, our research conclusions were consistent. Students who adopt positive coping styles tend to have better psychological resilience, better coping measures and more psychological support, while students who adopt negative coping styles are more likely to develop negative attitudes and even suicidal behaviors (31, 32).

This study experienced certain limitations: (1) as this study is just a cross-sectional study, longitudinal studies with large samples are needed to verify the above conclusions; (2) it was unclear whether the psychiatric or psychological conditions of the college students might influence their work and study; (3) the diagnosis of depression and anxiety is based on scales instead of clinical criteria. As there is an overlap of symptoms of anxiety or depression, it may result in overestimation and inaccuracy.

## Conclusions

During the epidemic of Omicron, a significant number of college students, especially boys, will suffer from anxiety or depression due to the closure of the school. Therefore, we should pay attention to the psychological state of this group of people, and we recommend the use of positive coping methods such as learning or study to prevent bad emotions during the isolation period.

## Data availability statement

The original contributions presented in the study are included in the article/supplementary material, further inquiries can be directed to the corresponding author.

## Ethics statement

The studies involving human participants were reviewed and approved by the Ethics Committee of Shanghai Jiao Tong University. The patients/participants provided their written informed consent to participate in this study. Ethical review and approval was not required for the animal study because the Ethics Committee of Shanghai Jiao Tong University.

## Author contributions

WL contributed to the study concept and design, analyzed the data, and drafted the manuscript.

## Funding

This study was supported by grants from the Clinical Research Center project of Shanghai Mental Health Center (CRC2017ZD02), Clinical Research plan of SHDC (SHDC2020CR1038B), the Cultivation of Multidisciplinary Interdisciplinary project in Shanghai Jiao Tong University (YG2019QNA10), Curriculum Reform of Medical College of Shanghai Jiao Tong University, the Feixiang Program of Shanghai Mental Health Center (2020-FX-03 and 2018-FX-05), the National Natural Science Foundation of China (82101564, 82001123), Chinese Academy of Sciences (XDA12040101), Shanghai Clinical Research Center for Mental Health (SCRC-MH, 19MC1911100), the Shanghai Science and Technology Committee (20Y11906800), and Shanghai Brain Health Foundation (SHBHF2016001).

## Conflict of interest

The author declares that the research was conducted in the absence of any commercial or financial relationships that could be construed as a potential conflict of interest.

## Publisher's note

All claims expressed in this article are solely those of the authors and do not necessarily represent those of their affiliated organizations, or those of the publisher, the editors and the reviewers. Any product that may be evaluated in this article, or claim that may be made by its manufacturer, is not guaranteed or endorsed by the publisher.
